# Transcriptome Analysis of *Ostrinia furnacalis* Female Pheromone Gland: Esters Biosynthesis and Requirement for Mating Success

**DOI:** 10.3389/fendo.2021.736906

**Published:** 2021-09-17

**Authors:** Shuangyan Yao, Shuai Zhou, Xiang Li, Xiaoguang Liu, Wenli Zhao, Jizhen Wei, Mengfang Du, Shiheng An

**Affiliations:** State Key Laboratory of Wheat and Maize Crop Science/College of Plant Protection, Henan Agricultural University, Zhengzhou, China

**Keywords:** *Ostrinia furnacalis*, PBAN, secondary messenger, signal transduction, sex pheromone

## Abstract

Female moths use sex pheromones to attract males, and corresponding regulatory mechanism underlying sex pheromone biosynthesis is species-dependent. However, the detailed mechanism involved in sex pheromone biosynthesis in *Ostrinia furnacalis* has not yet been fully addressed. In the present study, transcriptome sequencing of *O. furnacalis* pheromone glands screened a serials of candidate genes involved in sex pheromone biosynthesis. Our analysis showed that sex pheromone release in *O. furnacalis* females arrives its peak at the 2^nd^ scotophase, consistent with its mating behavior. Pheromone biosynthesis-activating neuropeptide (PBAN) was confirmed to regulate sex pheromone biosynthesis, and Ca^2+^ is the secondary messenger of PBAN signaling in *O. furnacalis*. The functional analysis of candidate genes demonstrated that the decreased mRNA levels or activities of calcineurin (CaN) and acetyl-CoA carboxylase (ACC) led to significant decrease in sex pheromone production and female capability to attract males, as demonstrated by RNAi-mediated knockdown and pharmacological inhibitor assay. Most importantly, the activities of CaN and ACC depend on the activation of PBAN/PBANR/Ca^2+^. Furthermore, fatty-acyl reductase 14 was involved in PBAN-mediated sex pheromone biosynthesis. Altogether, our results demonstrated that PBAN regulates sex pheromone biosynthesis through PBANR/Ca^2+^/CaN/ACC pathway to promote sex pheromone biosynthesis in *O. furnacalis* and provided a reference for non-model organism to study neuropeptide signal transduction.

## 1 Introduction

Sexual reproduction is the most important and universal mechanism for the proliferation of a population and survival of a species, especially in the animal kingdom. Being the largest and most abundant group in the animal kingdom, insects heavily rely on their complex and efficient reproductive process. In lepidopteran insects, sex pheromones are important mating agents that play a vital role in regulating mating success. Female moths usually emit species-specific pheromones that cause conspecific males to fly upwind in search of potential mates, thus facilitates the finding of a potential mate (1). Sex pheromones in Lepidoptera had been studied for half a century since the initial identification of bombykol from the silk moth *Bombyx mori* (2). So far, approximately 700 sex pheromone components had been identified from more than 1,600 species of moths (3) (https://www.pherobase.com/). Sex pheromones in female moths are classified into “type I,” “type II,” miscellaneous, “type 0,” and “type III” based on factors, such as functional group and carbon chain length (4, 5). Typical type I compounds have a functional group (such as an acetyl, hydroxyl, or aldehyde group) at the terminal position. Typical type II compounds are characterized by C17–C25 polyunsaturated (two to five double bonds) hydrocarbons and their corresponding epoxy derivatives (4). Type 0 pheromones are short-chain secondary alcohols, and their corresponding methyl ketones are obtained from primitive moths (6). Type III pheromones are carbon chains containing one or more methyl branches (5). Studies on the composition and structure of sex pheromone components have mainly contributed to the integrated pest management of moths. For example, sex pheromones have been widely used to control the codling moth *Cydia pomonella*, diamondback moth *Plutella xylostella*, gypsy moth *Lymantria dispar*, as well as other pests (7, 8).

In addition to their application in pest control, sex pheromones have also attracted the interest of researchers in basic biological fields, such as reproductive isolation, speciation, signal transduction, and hormonal control of production (9–12). Notably, several epoch-making findings have been made, such as the identification of pheromone biosynthesis-activating neuropeptide (PBAN), the regulator of sex pheromone biosynthesis and release (13, 14), and the identification of PBAN receptors (15). PBAN regulated sex pheromone biosynthesis in most moths. Initial structure-function analyses of *B. mori* PBAN highlighted the importance of the C-terminus in regulating pheromonotropic activity. Further studies revealed that a minimal pentapeptide sequence (Phe-Ser-Pro-Arg-Leu-NH_2_) is required for the pheromonotropic activity of PBAN (16, 17). Correspondingly, PBAN function has been well elucidated in many moths. PBAN is released into the hemolymph, where it binds to its receptor (PBANR) on the surface of PG cells. The binding of PBAN and PBANR leads to the conformational change of PBANR, and the influx of Ca^2+^. The concomitant rise in intracellular Ca^2+^ results in the formation of Ca^2+^-calmodulin complexes. The subsequent pathway exhibits species-dependent divergence. In *B. mori*, Ca^2+^-calmodulin complexes activate calcineurin (CaN) and calmodulin-dependent kinase II (CaMKII). Further, CaN catalyzes fatty acyl reductase (FAR) action, which is the final step of Bombykol biosynthesis (i.e., reduction of Δ10,12-hexadecadienoate to 10,12-hexadecadien-1-ol). Meanwhile, CaMKII phosphorylates lipid storage droplet protein-1, which leads to the lipolytic release of stored pheromone precursors from the cytoplasmic lipid droplets. In *Helicoverpa armigera*, CaN activated by Ca^2+^/CaM complexes dephosphorylates acetyl CoA carboxylase (ACC), the limiting step in fatty acid biosynthesis, thereby leading to sex pheromone biosynthesis. In addition to Ca^2+^, the binding of PBAN with PBANR in *H. armigera* stimulates adenylate cyclase activity and the production of cAMP, which, in turn, inhibits AMPK activity (the upstream kinase that inhibits ACC activity by dephosphorylation), thus ensuring the dephosphorylated status of ACC and subsequent sex pheromone biosynthesis (11, 18). These studies well addressed the molecular mechanism underlying PBAN-regulated sex pheromone biosynthesis.

Asian corn borer, *Ostrinia furnacalis*, was a serious pest that caused serious damage to economic crop maize in China, resulting in a 10–30% yield loss (19). Sex pheromones in *O. furnacalis* have been identified over the past 35 years (20). *O. furnacalis* females use Z12–14: OAc and E12–14: OAc in a ratio of 53:47 as the main sex pheromone components to attract males (21). Monitoring and large-scale trapping of *O. furnacalis* using sex pheromone bait traps has gradually become prevalent in China and other Asian regions (22–24). In addition to these applications, some studies have been conducted on sex pheromone biosynthesis. PBAN mediated sex pheromone biosynthesis in *O. furnacalis* by regulating the early step of transition from acetate to palmitic acid (25). Most importantly, PBANR has been identified from *O. furnacalis* (26). These results provide an important foundation for elucidating the molecular mechanisms underlying PBAN-mediated sex pheromone biosynthesis in *O. furnacalis*.

Most moths employ typical type I sex pheromones to attract males. Typical type I sex pheromones contain alcohols, aldehydes, and esters (4, 27, 28). The molecular mechanism underlying PBAN-regulated sex pheromone biosynthesis has been well elucidated in *B. mori* (utilize alcohol as a sex pheromone) and heliothine species (employ aldehydes as sex pheromone), such as *H. armigera.* However, the corresponding molecular mechanism in species that use acetate ester as sex pheromone was not well-elucidated. *O. furnacalis* was employed as a model in the present study to investigate sex pheromone biosynthesis to further decipher the molecular mechanism underlying PBAN-regulated biosynthesis using acetate ester as sex pheromone. These studies also provided a reference for non-model organism to study neuropeptide signal transduction.

## 2 Materials and Methods

### 2.1 Insect

Adult moths of *O. furnacalis* were collected in Jiyuan city, Henan Province, China (112.57°N, 124.46°E), and maintained in our laboratory for 1 year. Offspring were reared on an artificial diet at 28°C under a photoperiod of 14L and 10D with 60% relative humidity (29). Pupae were placed in 20×20×10 cm^3^ cage at 23–25°C, 60–70% relative humidity, and 14L: 10D photoperiod until emergence. Adults were fed with 5% sugar water.

### 2.2 Chemical

The PBAN-like peptide (Ser-Arg-Thr-Lys-Tyr-Phe-Ser-Pro-Arg-Leu-NH_2_) was synthesized by Sangon Biotech Company (Shanghai, China). *O. furnacalis* sex pheromone components, (E)-tetradec-12-enyl acetate (E12-14: OAC) and (Z)-tetradec-12-enyl acetate (Z12-14: OAC), were purchased from Sigma Company (St. Louis, MO, USA) and were used to quantify sex pheromone titer in the PGs by gas chromatography (GC). The 5-(tetradecyloxy)-2-furoic acid (TOFA, an ACC inhibitor), LaCl_3_ (a Ca^2+^ inhibitor), and tacrolimus (FK506, CaN-specific inhibitor) were purchased from Sigma (St. Louis, MO, USA).

### 2.3 PG Transcriptome Sequencing

To fully cover all genes involved in sex pheromone biosynthesis, PG at three different development time points [−48 h (48 h before emergence), 0 h (new emergence), and 48 h (48 h after emergence)] were collected. Each sample included at least 200 PGs for extracting enough total RNA. The samples were used by GENEWIZ (Suzhou, China) for transcriptome sequencing.

Total RNA was extracted from collected PG tissues following the protocol of Trizol (Invitrogen, Carlsbad, USA). DNase I (Invitrogen, Carlsbad, USA) was used to remove to genomic residuals. The quality (concentration and integrity) of total RNA was determined by Nanodrop ND-2000 spectrophotometer (NanoDrop, USA) and Agilent 2100 BioAnalyzer (Agilent, PaloAlto, USA). RNA seq library was constructed by using RNA-Seq Library Preparation Kit (Gnomegen, San Diego, USA) according to the manufacturer’s instructions. Library was sequenced by on Illumina HiSeqTM2000 platform (Illumina, San Diego, USA). Transcriptomic sequence has been submitted NCBI (accession number is PRJNA751717).

### 2.4 *De Novo* Assembly and Annotation of PG Transcript

The low-quality tags (tag sequences, adaptor sequences, and contaminated reads) were filtered, and clean tags were further assembled to contigs by using Trinity (30). The unigenes were generated by pooling contigs together. The assembled unigenes were further annotated by using NR, NT, KO, SwissProt, PFAM, GO, and KOG databases through BLASTX (E value ≤ 1e−5).

### 2.5 Sample Preparation

#### 2.5.1 PG Samples at Different Time Points

PGs were collected from female moths at different developmental time points [72 h before emergence (−72 h), 48 h before emergence (−48 h), 24 h before emergence (−24 h), new emergence (0 h), 24 h after emergence (24 h), 48 h after emergence (48 h), and 72 h after emergence (72 h)]. PGs from at least 30 females were used for total RNA extraction. Three biological replicates were chosen from each sample.

#### 2.5.2 Different Tissue Samples

Different tissues [head (HD), epidermis (EP), fat body (FB), muscle (MS), mid gut (MG), and PGs] were harvested from 48 h old females. Every sample contained at least 30 females and was used for total RNA extraction. Three biological replicates were used for each sample.

#### 2.5.3 PG Samples of Different Scotophase After Emergence

Newly emerged females were designated as 0 scotophase. PG samples were collected at 23:00 on each scotophase at 1^st^, 2^nd^, 3^rd^, 4^th^, and 5^th^ after emergence. Each sample contained at least 30 PGs. Collected PG samples were immersed in 150 μl of n-hexane for GC analysis. Three biological replicates were used.

#### 2.5.4 Transcriptional Level Determination

Total RNA was extracted using TRIzol (Invitrogen, Carlsbad, CA, USA). First-strand cDNA was synthesized from each RNA sample using a PrimeScript RT reagent kit (TaKaRa, Dalian, China) according to previously described methods (11, 31). The primers for qPCR analysis are listed in **Table S1**. The *actin* (GeneBank accession GU301782.1) and *18s* gene (GeneBank accession KT343378.1) were selected as reference genes (11). qRT-PCR was carried out using SYBR Green Supermix (TaKaRa, Dalian, China) on an Applied Biosysterms 7500 Fast Real-time PCR system (ABI, Carlsbad, CA, USA) according to the manufacturer’s instructions. The program was set according to report from Zhang et al. (32). The mRNA expression levels of genes were normalized with the two reference genes (*actin* and *EF-1α*) and analyzed with the DPS data processing system V7.05 (33).

### 2.6 Sample Treatment

#### 2.6.1 PBAN Treatment

Newly emerged females were decapitated and maintained for 24 h to deplete endogenous PBAN. Every female was injected with 10 pmol PBAN-like peptide and incubated at 28°C. PG tissues were collected at different time points of PBAN-like peptide injection(0, 30, 60, 90, and 120 min)and then dissolved in 150 μl of n-hexane followed by GC analysis. Correspondingly, PG tissues were collected at different time points after PBAN-like peptide injection(0, 30, 60, 90, and 120 min)and stored in −80°C refrigerator for subsequent enzyme activity assay. Three biological replicates were used, and each replicate contained at least 30 PGs.

#### 2.6.2 Double-Strand RNA Synthesis and Injection

ATP, CTP, GTP, and UTP (Thermo Fisher Scientific, MA, USA) were used to synthesize dsRNA. All of the primers were present in **Table S1**. The reaction system includes 10 μl 5*Transcription buffer, 1 μl ATP (100 mM), 1 μl CTP (100 mM), 1 μl GTP (100 mM), 1 μl UTP (100 mM), 1.25 μl RNA enzyme inhibitor, 3 μl T7 RNA Polymerase, and 1.5 μg template. Finally, DEPC H_2_O was used up to 50 μl. The reaction was carried out by incubating at 37°C for 4 h, and then the synthesized dsRNA was purified by using phenol chloroform and 3M sodium acetate.

The newly emerged females were decapitated and maintained in normal condition for 24 h. The 15 μg ds*CaN*/ds*ACC* was injected into decapitated females, and after 24 h waiting time, PG tissues were harvested and subjected to qRT-PCR analysis. The females injected with ds*EGFP* were used as controls. Three biological replicates were used, and each replicate contained at least 30 PGs.

The newly emerged females were decapitated and maintained in normal condition for 24 h. The 15 μg ds*CaN*/ds*ACC* was injected into decapitated females, and 24 h after injection, 10 pmol PBAN-like peptide was injected into treated females. After 1 h waiting time, PGs were collected for GC and enzyme activity analysis. Three biological replicates were used, and each replicate contained at least 30 PGs.

Newly emerged females were injected with 15 μg ds*CaN* or ds*ACC* and were maintained at normal condition for 48 h. The treated females were then used to test the ability to attract males. The females injected with ds*EGFP* were used as control.

#### 2.6.3 Inhibitor Treatment

Newly emerged females were decapitated and maintained for 24 h. PGs were then collected and further treated with 10 μM FK506 (CaN inhibitor) or 4 mM TOFA (ACC inhibitor) for 2 h followed by PBAN treatment for 60 min. The PG samples were harvested and subjected to GC and enzyme activity assay. PG samples treated with DMSO were used as control. Three biological replicates were used, and each replicate contained at least 30 PGs.

### 2.7 cAMP and Ca^2+^ Measurement

Newly emerged females were decapitated and maintained for 48 h to deplete endogenous PBAN. PGs were harvested and incubated with Grace’s insect cell medium for 2 h. PBAN-like peptide of 10 pmol was then added to medium. PG tissues were collected at different time points of PBAN-like peptide treatments (0, 30, 60, 90, and 120 min) and subjected to cAMP and Ca^2+^ measurement. cAMP was measured by insect cAMP ELISA Kit (LMAI, Shanghai, China) according to the manufacturer’s instructions. Ca^2+^ was measured by Calcium Assay Kit (Beyotime, Jiangsu, China) by o-cresolphthalein complexone colorimetry according to the manufacturer’s instructions. Three biological replications were used, and each replicate contained at least 30 PGs.

### 2.8 CaN and ACC Activity Analysis

The enzyme activity of CaN was assayed using a Calcineurin Activity Assay Kit (Jiancheng, Nanjing, China) according to previously described method (11). ACC enzyme activity was assayed using Acetyl CoA carboxylase Assy Kit (Jiemei, Nanjing, China), according to previously described method (11).

Three biological replicates were used, and each replicate contained at least 30 PGs.

### 2.9 GC Analysis

*O. furnacalis* sex pheromone components (Z12-14: OAC and E12-14: OAC) were measured using a GC instrument (Agilent7890B). The program was set as following: the temperature was maintained at 60°C for 2 min, increased to 180°C at 30°C/min, then to 230°C at 5°C/min, during which all the pheromone components were eluted. The column was then heated to 245°C at 20°C/min and held at this temperature for 15 min to clean the column before the next analysis. The FID detector was held at 250°C.

### 2.10 Mating Behavior

Females (n>20) at different developmental time points [0 h (newly emergence), 24 h (the first scotophase after emergence), 48 h (the second scotophase after emergence), 72 h (the third scotophase after emergence), 96 h (the fourth scotophase after emergence), and 120 h (the fifth scotophase after emergence)] were placed in six cages (40×40×40 cm), respectively. Correspondingly, 24 males (homogeneous, healthy and 48 h after emerged) were replaced in each cage. Females and males were kept together for approximately 24 h, and the proportion of successful copulatrix of females was assessed by determining the presence of a spermatophore in the female bursa copulatrix.

### 2.11 Female Ability to Attract Males

The attract device is shown in **Figure S1**. Females in treatment group (injection with ds*CaN* or ds*ACC*) and control group (injection with ds*EGFP*) were placed in lure cells respectively. Then 48 h old males were placed on released cell located at the top of the device so that males could freely choose the females below. Under the action of the atmospheric sampler, the flowing air transports the female sex pheromone from bottom to top, so that males could sense sex pheromone from females. After one night, the number of males that attracted by treatment females and control females were counted (32). All the experiments were repeated three times.

### 2.12 Statistical Analysis

QRT-PCR was used to investigate the spatial distribution of sex pheromone biosynthesis-related genes. Multiple comparison tests were conducted to determine the significant differences in mRNA expression level of sex pheromone biosynthesis-related genes at different tissues (*P <* 0.05, Tukey’s test, DPS7.05).

The effect of PBAN on sex pheromone was detected by GC method. The significant differences in sex pheromone titers at different time points of PBAN stimulation were compared using a multiple comparison (*P <* 0.05, Tukey’s test, DPS7.05).

Multiple comparison tests were conducted to determine the significant differences in the concentration of Ca^2+^ or cAMP at different time points of PBAN incubation (*P <* 0.05, Tukey’s test, DPS7.05).

Multiple comparison tests were conducted to determine the significant differences in the activity of CaN or ACC at different time points of PBAN treatments (*P <* 0.05, Tukey’s test, DPS7.05).

QRT-PCR was used to investigate RNAi efficacy. Significant difference in mRNA expression level of gene (PBANR, CaN, ACC, FAR-like, and FAR14) between *dsEGFP* injected females and double-strand target gene injected females was compared using Student’s *t*-test (* indicates *P <* 0.05, ** presents *P <* 0.01, and *** indicates *P <* 0.001).

The effect of RNAi on sex pheromone production was detected by GC method. The significant differences in sex pheromone production between *dsEGFP*-injected females and double-strand target gene injected females (PBANR, CaN, ACC, FAR-like, and FAR14) were compared using Student’s *t*-test (* indicates *P <* 0.05, ** presents *P <* 0.01, and *** indicates *P <* 0.001).

The effect of inhibitor (including LaCl_3_, FK506, or TOFA) on sex pheromone production was detected by GC method. The significant difference in sex pheromone production between inhibitor-treated PGs and control PGs were compared using by Student’s *t*-test (* indicates *P <* 0.05, *** indicates *P <* 0.001).

The effect of FK506 treatment on ACC activity was measured by using corresponding kit mentioned above. The significant differences in ACC activity between FK506-treated PGs and control PGs were compared using by Student’s *t*-test (** indicates *P <* 0.01).

The effect of RNAi-mediated knockdown of CaN on ACC activity was measured by using corresponding kit mentioned above. The significant differences in ACC activity between *dsCaN-*injected females and *dsEGFP*-injected females were compared using Student’s *t*-test (** indicates *P <* 0.01).

The female ability to attract males was investigated by recording the number of males attracted by female. The significant differences in female ability to attract males between treated and control females were compared using Student’s *t*-test (** indicates *P <* 0.01).

## 3 Results

### 3.1 Transcriptomic Sequencing and Assembly

The PG transcriptome was generated to explore the genes involved in sex pheromone biosynthesis of *O. furnacalis*. Transcriptomic sequencing of *O. furnacalis* PGs generated 39,306,428 raw sequencing reads (>5.9 Gb). Overall, 37,127,328 clean sequencing reads were obtained after filtering out noise signaling (containing N, low quality, and adapter related). Total unigenes with an average length of 814 bp, an N50 length of 1,619 bp, and an N90 length of 298 bp were obtained after Trinity assembly (**Table S2**).

A total of 33,139 unigenes were searched against the Nr database and annotated (**Table S3**). Further analysis revealed that 42 unigenes were associated with sex pheromone biosynthesis (**Table S4**). Homologous searches identified eight genes that serve as potential regulators of sex pheromone biosynthesis. These genes included protein kinase A (*PKA*), protein kinase C delta (*PKCδ*), Δ14-desaturase (*Des-14*), PBAN receptor (*PBANR*), *CaN*, *ACC*, fatty acid reductase (*FAR-like*), and fatty acid reductase 14 (*FAR14*) (**Table S4**).

### 3.2 Spatial Distribution of Sex Pheromone Biosynthesis-Related Genes

The spatial distributions of the eight genes mentioned above were investigated in different tissues (HD, EP, FB, MS, MG, and PG). Results revealed that *PBANR* (F = 2197.7, df = 17, *P* = 0.0001), *Des-14* (F = 401.9, df = 17, *P* = 0.0001), *FAR-like* (F = 4654.7, df = 17, *P* = 0.0001), and *FAR14* (F = 397.7, df = 17, *P* = 0.0001) were specifically expressed in PGs (**Figures S2A, F–H**). *PKA* (F = 31.0, df = 17, *P* = 0.0001), which peaked in FB, followed by MS and MG, was ubiquitously expressed in all examined tissues (**Figure S2B**). The expression level of *PKCδ* (F = 811.1, df = 17, *P* = 0.0001) was much higher in MS than in other tissues (**Figure S2C**). Similar to that of *PKA*, *CaN* and *ACC* were ubiquitously expressed and distributed in all examined tissues. The expression levels of *CaN* (F = 45.5, df = 17, *P* = 0.0001) and *ACC* (F = 958.3, df = 17, *P* = 0.0001) were the highest in FB (**Figure S2D**) and MG (**Figure S2E**). These results suggested that these genes play certain roles in these tissues.

### 3.3 Developmental Expression Patterns of Sex Pheromone Biosynthesis-Associated Genes

Developmental expression patterns of sex pheromone biosynthesis-associated genes were investigated using qRT-PCR. Results demonstrated that the expression level of *PBANR* rapidly increased before emergence and reached its highest level before emergence and new emergence (**Figure S3A**). *PKA* transcript exhibited a stable expression pattern (**Figure S3B**). *PKCδ* and *CaN* showed similar expression patterns, which began to express at 72 h before emergence and continued to increase and peaked at 48 h after emergence (**Figures S3C, D**). *ACC*, *Des-14*, and *FAR-like*, which are development-dependent, shared similar expression patterns (**Figures S3E–G**). The expression level of *FAR14* was higher after emergence than that before emergence (**Figure S3H**). The developmental expression patterns of sex pheromone biosynthesis-associated genes suggested their possible functions during PG development.

### 3.4 Fluctuation Rhythm of Sex Pheromone Release and Mating Frequency

In order to clarify the sex pheromone release pattern of *O. furnacalis* after emergence and provide a clear sampling time for subsequent experiments, sex pheromone titers were assayed and female mating frequency was counted. The main component of sex pheromone in *O. furnacalis*, Z12-14: OA, could be detected from new emergence, peaked at the 2^nd^ scotophase after emergence, remained at a high level at the 3^rd^ scotophase, and then decreased to base level (F = 286.7, df = 17, *P* = 0.0001) (**Figure S4A**). Consistently, mating frequency showed a pattern similar to the fluctuating pattern of Z12-14: OAC production (F = 70.3, df = 17, *P* = 0.0001) (**Figure S4B**). These results indicated that mating tests and attractant experiments should be conducted during the 2^nd^ scotophase.

### 3.5 Effect of PBAN Treatment on Sex Pheromone Production in *O. furnacalis*

**Figure 1:**
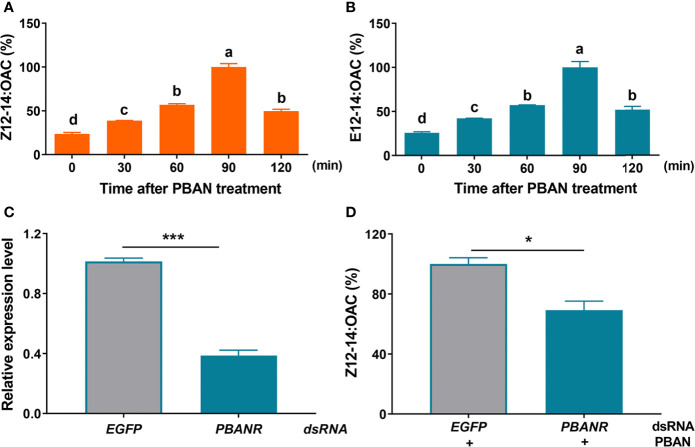
PBAN signal mediates sex pheromone production in *O. furnacalis*. **(A, B)** The titer of sex pheromone in PGs after PBAN incubation. Data represent the mean ± SE (n = 3). A multiple comparison test with *P <* 0.05 was conducted to test the significant differences in different development time points (based on Tukey’s test, DPS7.05). **(C)** RNAi efficiency of PBANR. **(D)** The effect of ds*PBANR* knockdown on sex pheromone production. Data represent the mean ± SE (n = 3). Statistically significant difference was denoted with * (*P <* 0.05) and *** (*P <* 0.001) as determined by Student’s *t*-test. Small letters a-d indicate significant difference.

To investigate the effect of PBAN on sex pheromone biosynthesis in *O. furnacalis* females, the titers of *O. furnacalis* sex pheromones, Z12-14: OAC and E12-14: OAC were measured in response to PBAN using GC analysis. The results revealed that PBAN treatment led to a significant increase in sex pheromone production (**Figures 1A, B**) (as for Z12-14: OAC, F = 178.0, df = 14, *P* = 0.0001; as for E12-14: OAC, F = 64.9, df = 14, *P* = 0.0001), indicating PBAN regulation of sex pheromone biosynthesis in *O. furnacalis* moths. Further knockdown of PBAN receptor (*P* = 0.0001) confirmed that RNAi-mediated knockdown of PBANR caused a significant reduction in sex pheromone production (*P* = 0.0256) (**Figures 1C, D**). These results verified that PBAN regulated sex pheromone biosynthesis through its receptor.

### 3.6 Measurement of the Second Messenger in PBAN Signal

**Figure 2:**
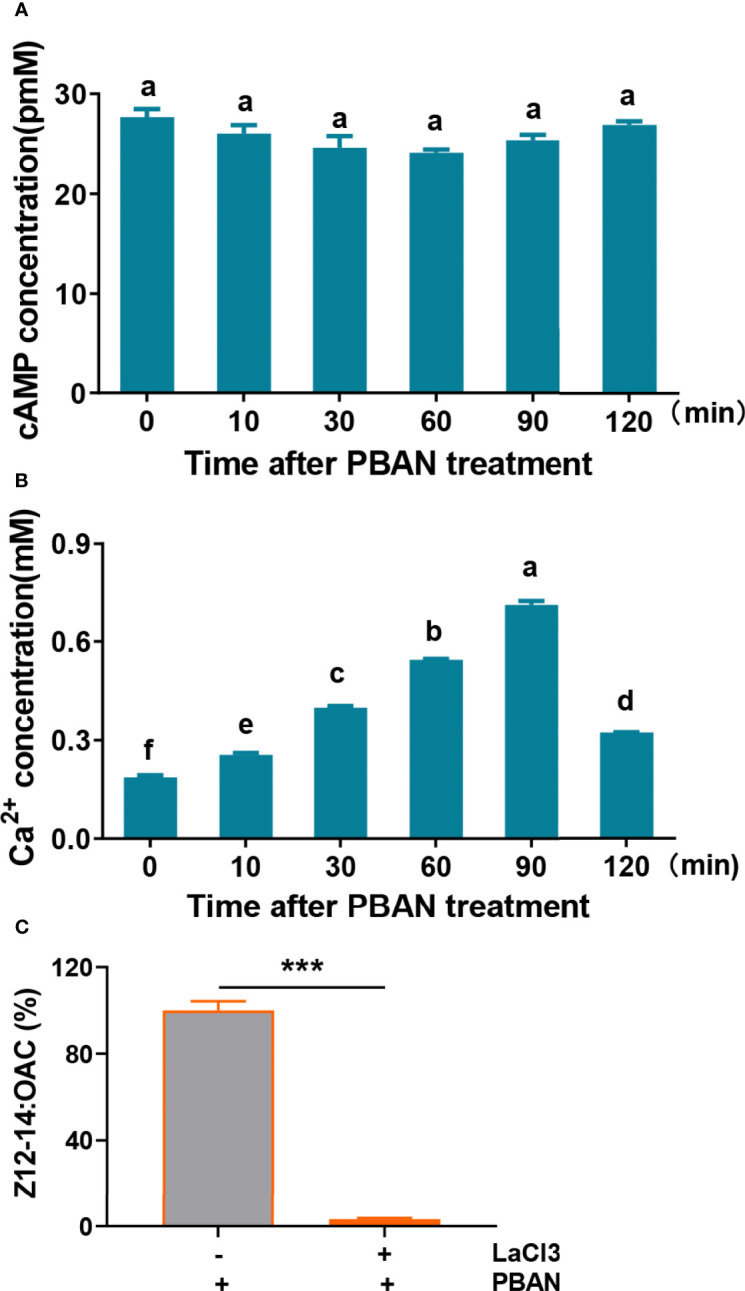
Second messengers of PBAN signal in *O. furnacalis*. **(A)** cAMP level in PGs after PBAN challenge in *O. furnacalis*. **(B)** The concentration of Ca^2+^ in PGs after PBAN incubation in *O. furnacalis*. Data represent the mean ± SE (n = 3). A multiple comparison test with *P <* 0.05 was conducted to test the significant differences in different development time points (based on Tukey’s test, DPS7.05). **(C)** Effect of the calcium channel inhibitor LaCl_3_ on sex pheromone production in *O. furnacalis* PGs. Data represent the mean ± SE (n = 3). Statistically significant difference was denoted with *** (*P <* 0.001) as determined by Student’s *t*-test. Small letters a-f indicate significant difference.

Both cAMP and Ca^2+^ are the most important second messengers in PBAN-regulated sex pheromone biosynthesis in Lepidoptera (11, 34). The levels of cAMP and Ca^2+^ were assayed in response to PBAN stimulation. Results demonstrated that PBAN stimulation did not influence cAMP levels (F = 1.9, df = 17, *P* = 0.1654) (**Figure 2A**). However, Ca^2+^ influx rapidly increased in response to the PBAN challenge, and reached its peak at 90 min after PBAN-like peptide incubation (F = 2610.4, df = 17, *P* = 0.0001) (**Figure 2B**). To further explore the effect of Ca^2+^ in PBAN signal, LaCl_3_, which will block calcium channels, was employed to investigate the effect of LaCl_3_ on sex pheromone production. Results showed that LaCl_3_ treatment caused a significant decrease in Z12-14: OAC production (*P* = 0.0010) (**Figure 2C**). These results revealed that PBAN employed Ca^2+^ as a second messenger to regulate sex pheromone biosynthesis.

### 3.7 Effect of PBAN on CaN and ACC Activity

**Figure 3:**
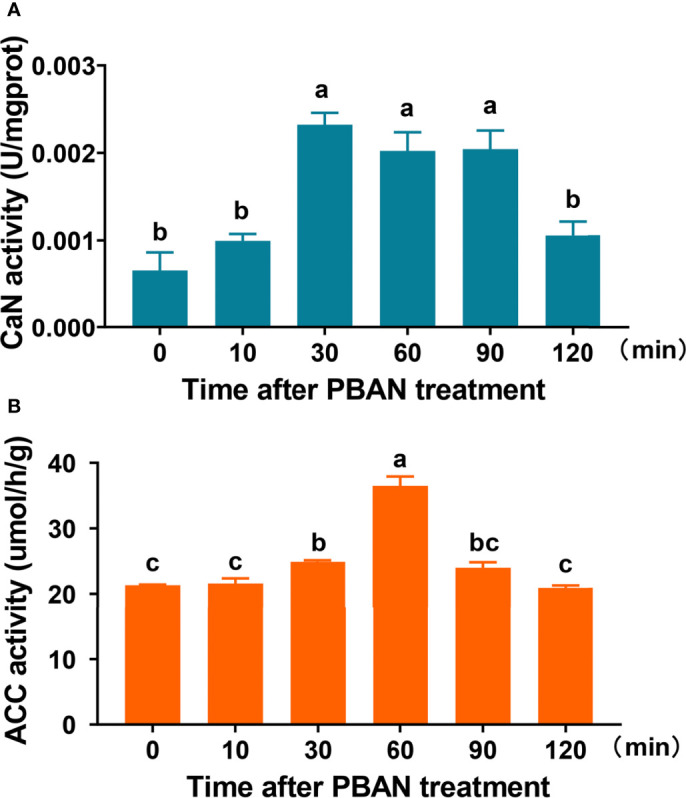
PBAN regulated CaN and ACC activity in *O. furnacalis* PGs. **(A, B)** The activity of CaN and ACC after PBAN incubation in *O. furnacalis* PGs. Data represent the mean ± SE (n = 3). A multiple comparison test with *P <* 0.05 was conducted to test the significant differences in different development time points (based on Tukey’s test, DPS7.05). NS, not significant. Small letters a-c indicate significant difference.

CaN and ACC activities in response to PBAN treatment were determined to investigate the effect of PBAN. CaN activity significantly increased 30 min after PBAN-like peptide treatment, remained at high levels 60 and 90 min after PBAN treatment, and finally decreased 120 min after PBAN treatment (F = 15.6, df = 17, *P* = 0.0001) (**Figure 3A**). Similarly, ACC activity increased 30 min after PBAN treatment, peaked at 60 min after PBAN treatment, finally declined to basal level (F = 57.9, df = 17, *P* = 0.0001) (**Figure 3B**). These results revealed that CaN and ACC were response to PBAN stimulation.

### 3.8 Effect of CaN on *O. furnacalis* Sex Pheromone Biosynthesis and Female Ability to Attract Males

**Figure 4:**
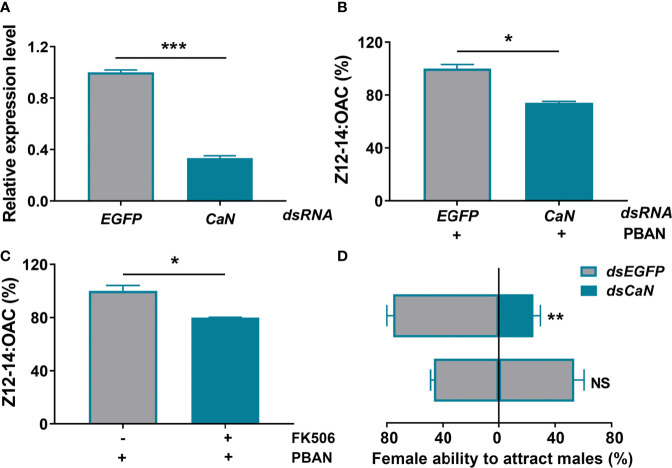
CaN positively regulates sex pheromone biosynthesis in *O. furnacalis* PGs. **(A)** The effects of dsRNA injection on the expression of CaN transcript. **(B)** Effect of ds*CaN* knockdown on sex pheromone production in *O. furnacalis* PGs. **(C)** Effect of the CaN inhibitor FK506 on sex pheromone production in *O. furnacalis* PGs. **(D)** Effect of ds*CaN* knockdown on female capability to attract males. Data represent the mean ± SE (n = 3). Statistically significant differences were denoted with * (*P <* 0.05), ** (*P <* 0.01), and *** (*P <* 0.001) as determined by Student’s *t*-test. NS, not significant.

RNAi was used to explore the role of CaN in sex pheromone biosynthesis in *O. furnacalis*. A ds*CaN* injection caused a significant decrease in CaN transcriptional level (*P <* 0.0001) (**Figure 4A**). The effect of CaN knockdown on sex pheromone production was investigated after successful transcript knockdown. Results demonstrated that RNAi-mediated knockdown of CaN led to a significant decrease in sex pheromone production (*P* = 0.0153) (**Figure 4B**). Similarly, the inhibition of CaN activity with a specific inhibitor (FK506) also caused a significant decrease of Z12-14: OAC production (*P* = 0.0195) (**Figure 4C**). The effect of CaN on female ability to attract males was also investigated. Results demonstrated that, compared with control (injected ds*EGFP*), the females that injected ds*CaN* attracted a smaller number of males (*P* = 0.0051) (**Figure 4D**), which was in agreement with sex pheromone biosynthesis.

### 3.9 Effect of ACC on *O. furnacalis* Sex Pheromone Biosynthesis and Ability to Attract Males

**Figure 5:**
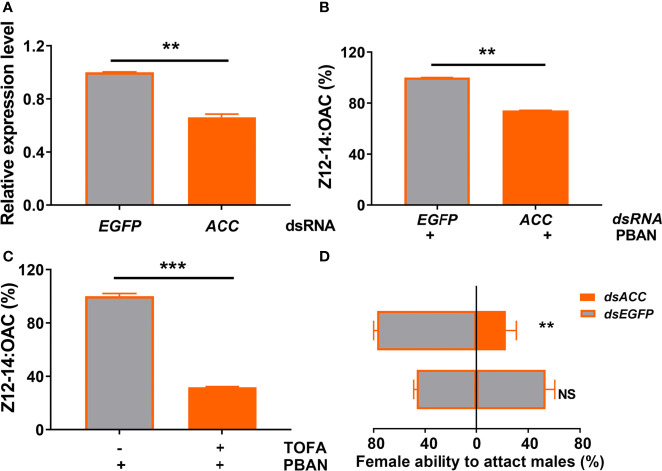
ACC positively regulates sex pheromone biosynthesis in *O. furnacalis* PGs. **(A)** The effects of dsRNA injection on the expression of ACC transcript. **(B)** Effect of ds*ACC* knockdown on sex pheromone production in *O. furnacalis* PGs. **(C)** Effect of the ACC inhibitor TOFA on sex pheromone production in *O. furnacalis* PGs. **(D)** Effect of ds*ACC* knockdown on female capability to attract males. Data represent the mean ± SE (n = 3). Statistically significant differences were denoted with ** (*P <* 0.01) and *** (*P <* 0.001) as determined by Student’s *t*-test. NS, not significant.

RNAi and inhibitor analyses of ACC were used to further investigate the effect of ACC on sex pheromone biosynthesis and female ability to attract males. qRT-PCR results showed that the mRNA level of ACC was significantly decreased after ds*ACC* injection (*P* = 0.0017) (**Figure 5A**). RNAi-mediated knockdown of ACC led to a significant decrease of sex pheromone (Z12-14: OAC) production, compared with the control injected with ds*EGFP* (*P* = 0.0037) (**Figure 5B**). Similarly, TOFA treatment (ACC inhibitor) resulted in a significant reduction in sex pheromone production (*P* = 0.0009) (**Figure 5C**). Most importantly, ACC knockdown in females significantly weakened their ability to attract males, compared to the controls injected with ds*EGFP* (*P* = 0.0028) (**Figure 5D**).

### 3.10 Effect of CaN on ACC Activity

**Figure 6:**
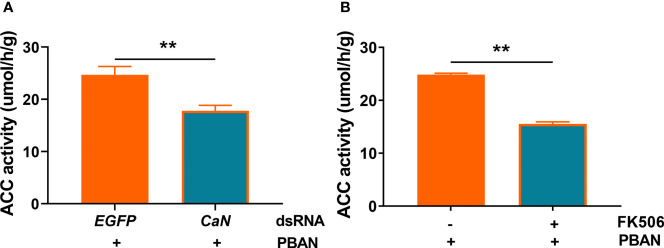
CaN positively regulates ACC enzyme activity in *O. furnacalis* PGs. **(A)** Effect of ds*CaN* knockdown on ACC enzyme activity in *O. furnacalis* PGs. **(B)** Effect of the CaN inhibitor FK506 on ACC enzyme activity in *O. furnacalis* PGs. Data represent the mean ± SE (n = 3). Statistically significant differences were denoted with ** (*P <* 0.01) as determined by Student’s *t*-test.

To explore the effect of CaN on ACC activity in response to PBAN signaling, RNAi-mediated knockdown of CaN or FK506-mediated inhibition of CaN activity was assayed. Results demonstrated that CaN knockdown significantly reduced ACC activity (*P* = 0.0014) (**Figure 6A**). Similarly, CaN inhibition also significantly reduced ACC activity (*P* = 0.0027) (**Figure 6B**). These results indicated that the activation of CaN by PBAN/PBANR/Ca^2+^ in turn activates ACC activity and, therefore, regulates sex pheromone biosynthesis.

### 3.11 Effect of FAR on *O. furnacalis* Sex Pheromone Production

To explore the function of FAR in *O. furnacalis* PGs, *FAR-like* and *FAR14* dsRNA were injected into females. The dsRNA injection led to a significant decrease in the mRNA expression levels of *FAR-like* (*P* = 0.0009) and *FAR14* (*P* = 0.0111) (**Figure S5A**). *FAR-like* knockdown did not affect sex pheromone levels; however, *FAR14* knockdown caused a significant decrease in sex pheromone production (**Figure S5B**) (*FAR-like*, *P* = 0.0597; *FAR14*, *P* = 0.0162), indicating *FAR14* is crucial in *O. furnacalis* sex pheromone biosynthesis.

## 4 Discussion

Most sex pheromones usually consist of hydrocarbons derived from acetyl-CoA through fatty acid synthesis followed by modification of carbonyl carbon, such as desaturation, reduction, and oxidase reaction (27). Correspondingly, many genes regulate the process of sex pheromone biosynthesis. For example, ACC catalyzed the conversion of acetyl CoA to 14, 16, and 18 carbon saturated fatty acids, which are sex pheromone precursors (35). Desaturases catalyzed the formation of double bonds in pheromone precursors, such as Δ5-desaturase (36), Δdesaturase (37), Δ9-desaturase (38, 39), Δ10-desaturase (40), Δ11-desaturase (39, 41, 42), and Δ14 -desaturase (43, 44). In addition, aldehyde reductase, FAR, and acetyltransferase have been used to modify the carbon chain to produce sex pheromones, such as alcohols, aldehydes, and acetate esters (28, 39, 45, 46). However, the corresponding gene sequences were unavailable because of the absence of *O. furnacalis* genomic information. Thus, screening associated genes involved in *O. furnacalis* sex pheromone biosynthesis is necessary to investigate the detailed underlying mechanism.

6-Although sex pheromones play vital roles in species-specific mating, the regulatory mechanism underlying sex pheromone biosynthesis is species-dependent. Juvenile hormone in female *Blattella germanica* regulates sex pheromone production (47). Similar results were also found in coleopteran insects, for instance, *Tenebrio molitor* (Tenebrionidae) (48–51), *Dendroctonus brevicomis* (52, 53), *Ips typographus* (54), *Pityokteines curvidens*, *P. vorontzovi* (55), *Scolytus scolytu*s (56), and *Tribolium castaneum* (Tenebrionidae) (57), in which juvenile hormones or its analogs regulate pheromone biosynthesis and/or release. Interestingly, ecdysteroids have also been shown to regulate sex pheromone production in Diptera. For example, ecdysteroid treatment induces the biosynthesis of sex pheromones in *Musca domestica* (58), *Sarcophaga bullata* (59, 60), and *Drosophila melanogaster* (61). In addition, PBAN has been confirmed to regulate sex pheromone biosynthesis in almost all Lepidoptera species. However, studies have demonstrated that PBAN does not stimulate sex pheromone biosynthesis in some species, such as *Trichoplusia ni* (62) and *Scoliopteryx libatrix* (63). These results indicated that the regulatory mechanism underlying sex pheromone biosynthesis is different even in lepidopteran moths. In the present study, PBAN treatment induces sex pheromone production in *O. furnacalis*. Most importantly, significant decrease of sex pheromone production due to RNAi-mediated knockdown of PBANR further verified the results of PBAN treatment. These results demonstrated that PBAN acts as a regulator in the regulation of pheromone production in *O. furnacalis*.

6-Numerous studies have been carried out to elucidate the molecular mechanism underlying the conversion of the external PBAN signal into a biological response and ultimately facilitating sex pheromone biosynthesis and release. Usually, PBAN binding to its G-coupled protein receptor triggers a series of signal transduction cascades that use a Ca^2+^ influx to activate enzymatic steps and generate sex pheromones. For example, PBAN stimulation in heliothine moths and *B. mori* caused a significant increase in intracellular Ca^2+^ (64). Moreover, studies have revealed that extracellular Ca^2+^ is essential for the pheromonotropic effect of PBAN in all examined moths, which elucidates the importance of Ca^2+^ in the PBAN signal transduction cascade. In the present study, Ca^2+^ influx rapidly increased in response to PBAN challenges in isolated *O. furnacalis* PGs. Most importantly, treatment with LaCl_3_, a pharmacological inhibitor of calcium channels, caused a significant decrease in sex pheromone production in *O. furnacalis*. This finding is consistent with the those of previous studies, indicating the conservation of Ca^2+^ as the secondary messenger of PBAN in moths. In addition to Ca^2+^, cAMP was also involved in the PBAN-signal transduction cascade. Studies have shown that PBAN stimulation led to a significant increase in cAMP levels in isolated *H. armigera* PGs (65, 66). Furthermore, the increase in PG cAMP levels by pharmacological treatment (cAMP analogs, adenylate cyclase activation) promoted sex pheromone biosynthesis and release in *H. armigera* (65, 66), *H. zea* (34), *H. virescens* (67), and *Argyrotaenia velutinana* (68), indicating that PBAN uses Ca^2+^ and cAMP as secondary messengers in these species. However, the role of cAMP in the PBAN signaling cascade appears to be species-dependent. Studies have confirmed that PBAN treatment does not lead to cAMP elevation in isolated PGs from *B. mori* (64), *S. litura* (69), and *O. nubilalis* (70), indicating that the role of cAMP in the PBAN signal cascade is species-dependent. Our present study also revealed that PBAN does not use cAMP as a secondary messenger in *O. furnacalis*, as demonstrated by the cAMP assay. Instead of cAMP, this condition indicates that Ca^2+^ was involved in the PBAN signal transduction cascade in these species. PBAN signaling cascade in *O. furnacalis* is similar to those in *B. mori* (64), *S. litura* (69), and *O. nubilalis* (70).

The different second messenger in the PBAN signal transduction cascade directly leads to the difference in the regulatory steps of pheromone biosynthesis. In species that do not undergo cAMP elevation, PBAN regulates a step(s) of fatty acyl reduction (70–73) and lipolysis step of cytoplasmic lipid droplet (74, 75). For example, in *B. mori*, CaN and CaMKII were activated in response to the rise of Ca^2+^ influx. Consequently, CaN regulated the step of reduction reaction catalyzed by FAR, 6-while CaMKII phosphorylated-activated lipid storage droplet protein-1, which led to the lipolytic release of stored pheromone precursors from the cytoplasmic lipid droplets. In species that utilize cAMP, PBAN regulates the biosynthetic pathway of fatty acids, most likely ACC (34, 62, 76). For example, a study demonstrated that PBAN regulated the ACC as the limiting step in *H. armigera* (76). The influence of PBAN on ACC enzyme activity was also found in *Argyrotaenia velutinana* (62), *P. interpunctella* (76), and *H. zea* (34). Further studies showed that CaN induced by Ca^2+^/CaM complexes dephosphorylated-activated ACC, thus promoting sex pheromone biosynthesis. In addition, activated cAMP/PKA led to suppression of the AMPK activity (the upstream kinase that inhibits ACC activity by dephosphorylation), which ensured the dephosphorylation of ACC and subsequent sex pheromone biosynthesis (10, 18). Our present study revealed that PBAN regulates sex pheromone biosynthesis in *O. furnacalis* through the Ca^2+^/CaN/ACC pathway, as demonstrated by RNAi-mediated knockdown and pharmacological inhibitor assay. Interestingly, PBAN signal transduction cascade in *O. furnacalis* copies that of *B. mori*, that is, PBAN only utilizes Ca^2+^ as secondary messenger. However, the regulatory step is similar to that of *H. armigera*, which exhibits the specificity of the mechanism of PBAN-regulated sex pheromone. However, the detailed mechanism requires further elucidation in future studies.

6-FAR catalyzes the conversion of the fatty-acyl precursors into the corresponding fatty alcohols and plays an important role in regulating sex pheromone biosynthesis and release. The functional identification has been shown in various moth species such as *B. mori* (77, 78), *Ostrinia nubilalis* (79), S*podoptera exigua* (80), and four *Heliothine* species (81). Interestingly, studies have demonstrated that multiple FARs in moths functioned in the conversion of specific unsaturated fatty acid precursors to the corresponding alcohols in the sex pheromone biosynthesis pathway (82) despite the different substrate preferences of these FARs. For example, in *B. mori*, a pgFAR showed substrate specificity for the bombykol precursor fatty acids. However, a signal pgFAR exhibited a broad substrate range in *Agrotis segetum* by catalyzing three precursors to their corresponding alcohols (83). Interestingly, two FARs showed different substrate specificities in *Spodoptera exigua*, one for C14 acyl-coA and other for C16 acyl-coA. In addition, different FARs were used to regulate sex pheromone biosynthesis, for example, Bm-pgFAR from *B, mori* (72), FAR2 from *H. armigera* (18), FAR3 from *S. litura* (84), and *FAR2* from *S. inferens* (85). In the present study, two FARs were screened from transcriptome data of *O. furnacalis* PGs. Further studies demonstrated that FAR14 regulates sex pheromone biosynthesis, indicating its role in sex pheromone biosynthesis of *O. furnacalis*.

**Figure 7:**
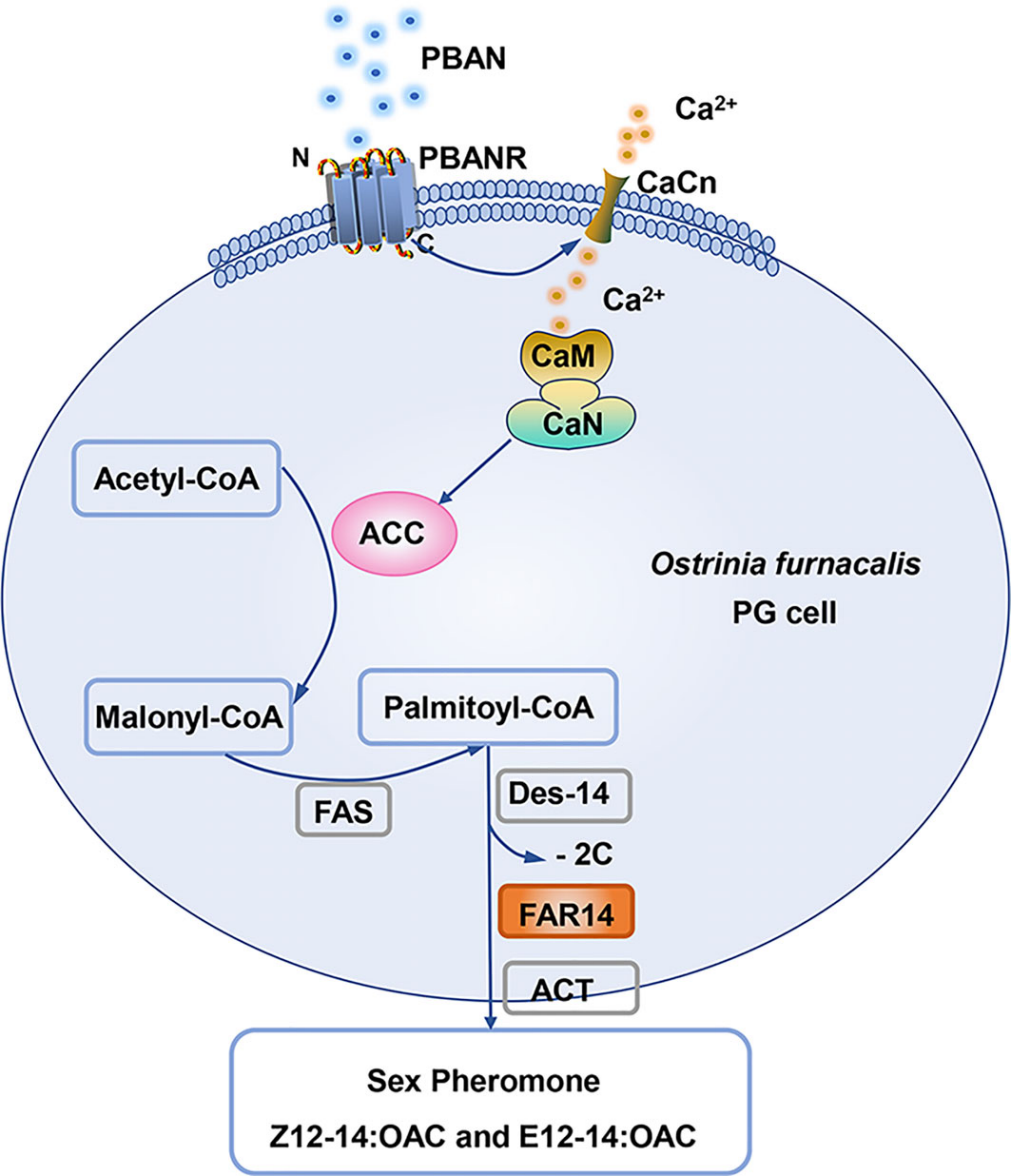
The model of PBAN signal transduction pathway in *O. furnacalis* PGs. The binding of PBAN with its receptor PBANR activates the second messenger (Ca^2+^). Ca^2+^ influx in turn activates CaN and ACC and ultimately regulates the production of sex pheromones under the catalysis of FAR14. PBAN, pheromone biosynthesis-activating neuropeptide; PBANR, PBANR receptor; CaCn, calcium channel; CaM, calmodulin; CaN, Calcineurin; ACC, Acetyl CoA carboxylase; FAS, fatty acid synthase; Des-14, delta 14 desaturase; FAR14, fatty acyl reductases 14; ACT, acyl transferase.

In summary, a model for PBAN-regulated sex pheromone biosynthesis was proposed in *O. furnacalis*, in which PBAN utilizes its secondary messengers (calcium ions) to regulate sex pheromone biosynthesis. Ca^2+^-activated CaN triggers ACC activity in response to the PBAN signal, thereby stimulating sex pheromone biosynthesis (**Figure 7**).

## Data Availability Statement

The datasets presented in this study can be found in online repositories. The names of the repository/repositories and accession number(s) can be found below: https://www.ncbi.nlm.nih.gov/bioproject/?term=prjna751717, PRJNA751717.

## Author Contributions

SY, validation, writing—original draft, and data analysis. SZ, experimentation. XL, software. XGL, supervision and conceptualization. JW, methodology. MD, supervision and conceptualization. SA, final draft and project administration. All authors contributed to the article and approved the submitted version.

## Funding

6-The work was supported by National Science fund of Henan Province for Distinguished Young Scholar (202300410191), the Basic Research Project of the Key Scientific Research Projects of Universities in Henan Province (21zx013), Henan Agricultural Research System (Grant S2014-11-G06), and Science and Technology Project in Henan Province (212102110463).

## Conflict of Interest

The authors declare that the research was conducted in the absence of any commercial or financial relationships that could be construed as a potential conflict of interest.

## Publisher’s Note

All claims expressed in this article are solely those of the authors and do not necessarily represent those of their affiliated organizations, or those of the publisher, the editors and the reviewers. Any product that may be evaluated in this article, or claim that may be made by its manufacturer, is not guaranteed or endorsed by the publisher.
