# Supplementary figures and images for: Transcriptome Analysis of Ostrinia furnacalis Female Pheromone Gland: Esters Biosynthesis and Requirement for Mating Success
Source: Front Endocrinol (Lausanne). 2021 Sep 17;12:736906. doi: 10.3389/fendo.2021.736906 (PMC8485726; doi:10.3389/fendo.2021.736906)

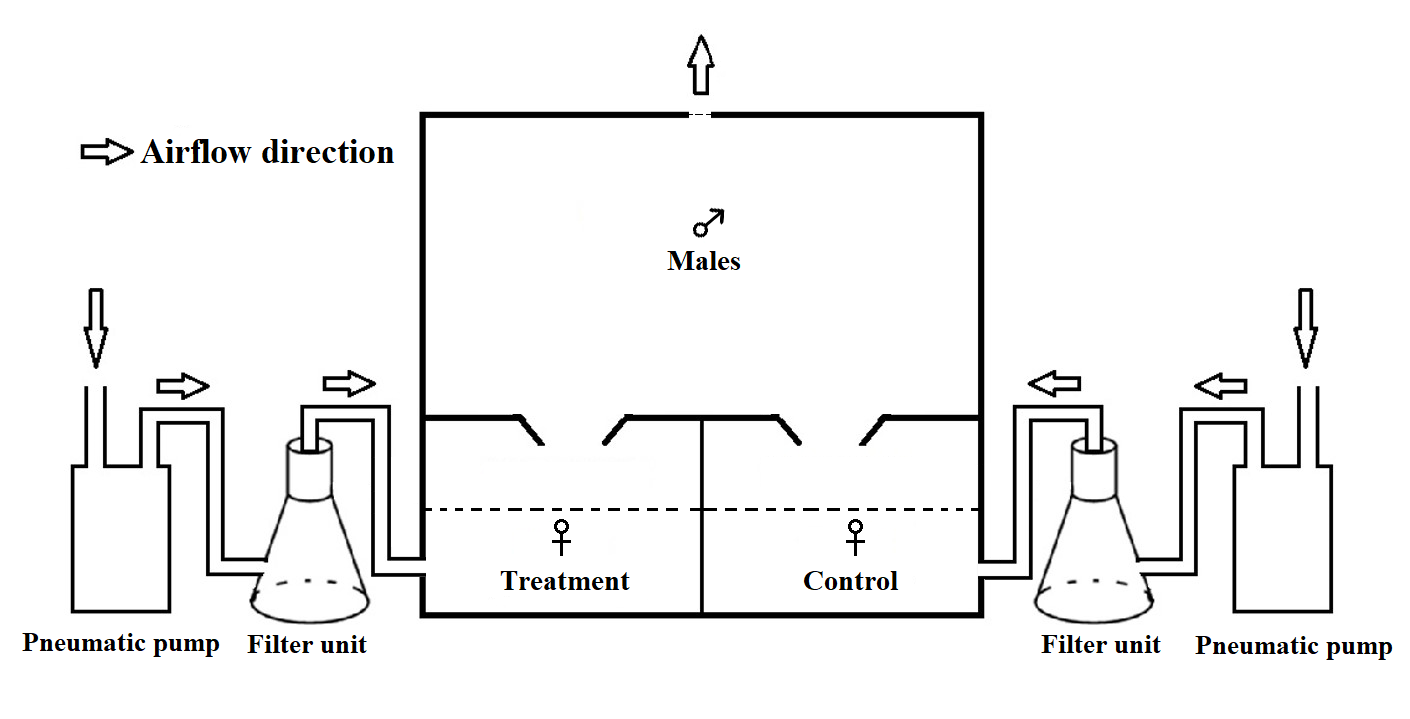

Supplement: Supplementary file 2 [file Image_1.tif]

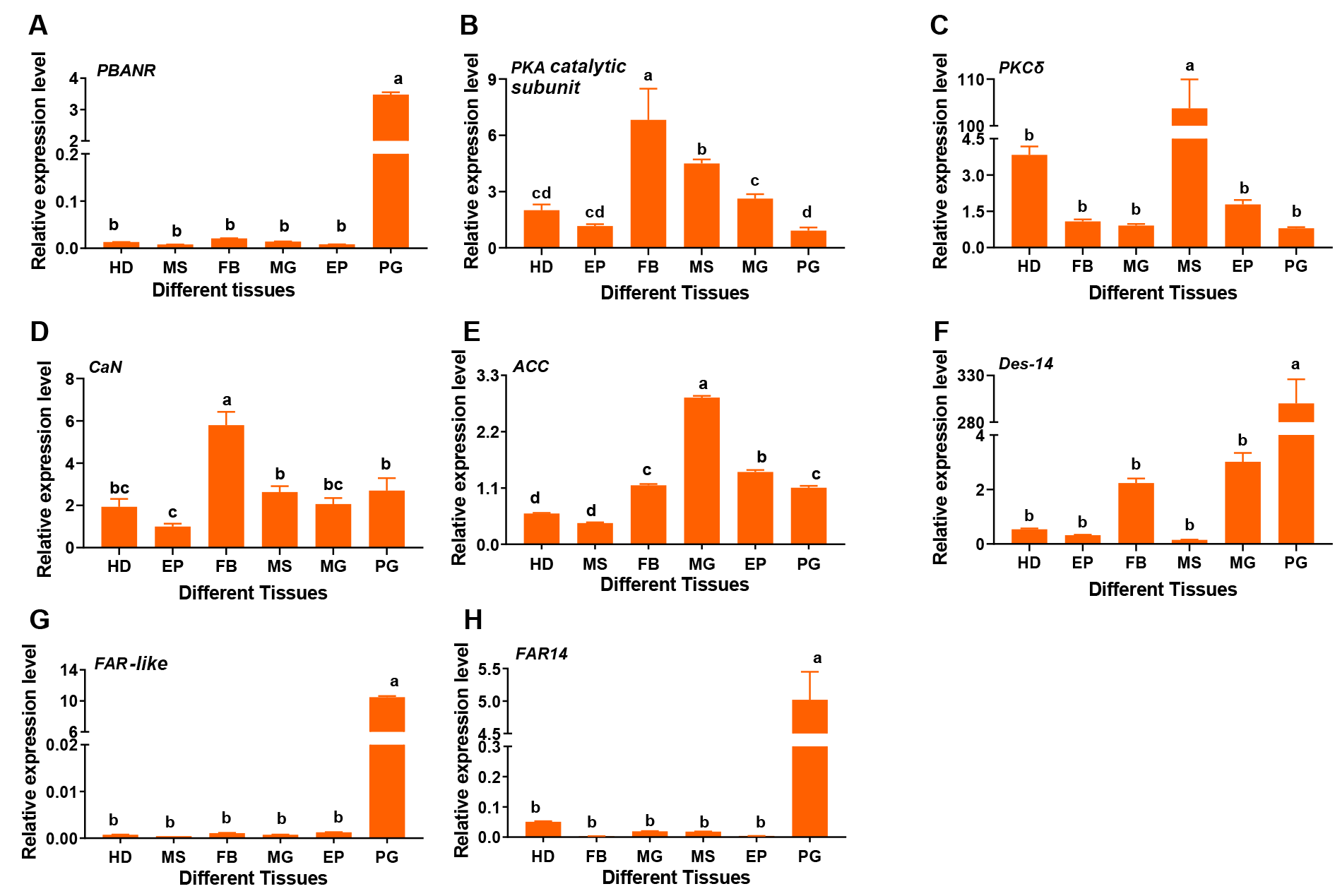

Supplement: Supplementary file 3 [file Image_2.tif]

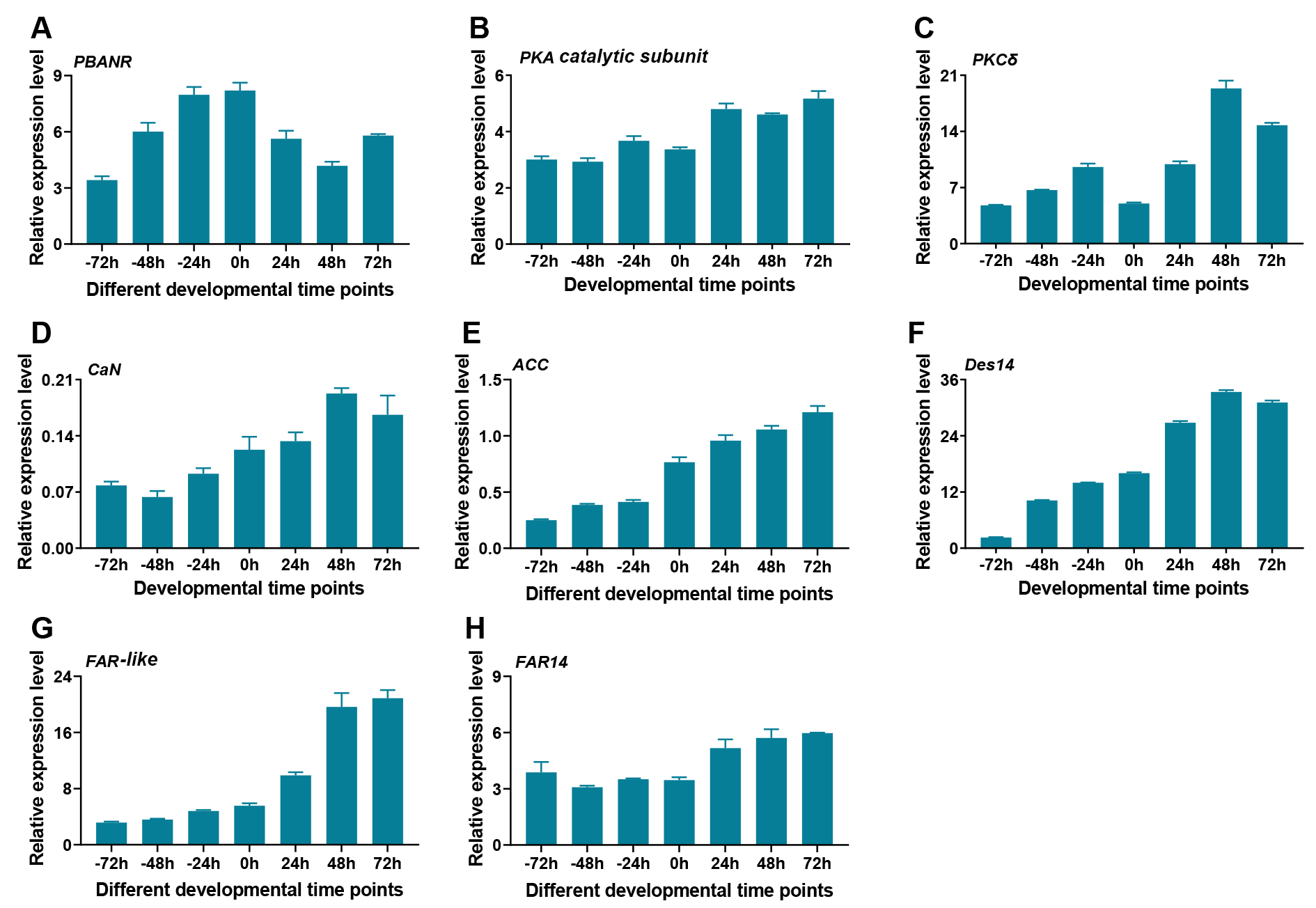

Supplement: Supplementary file 4 [file Image_3.tif]

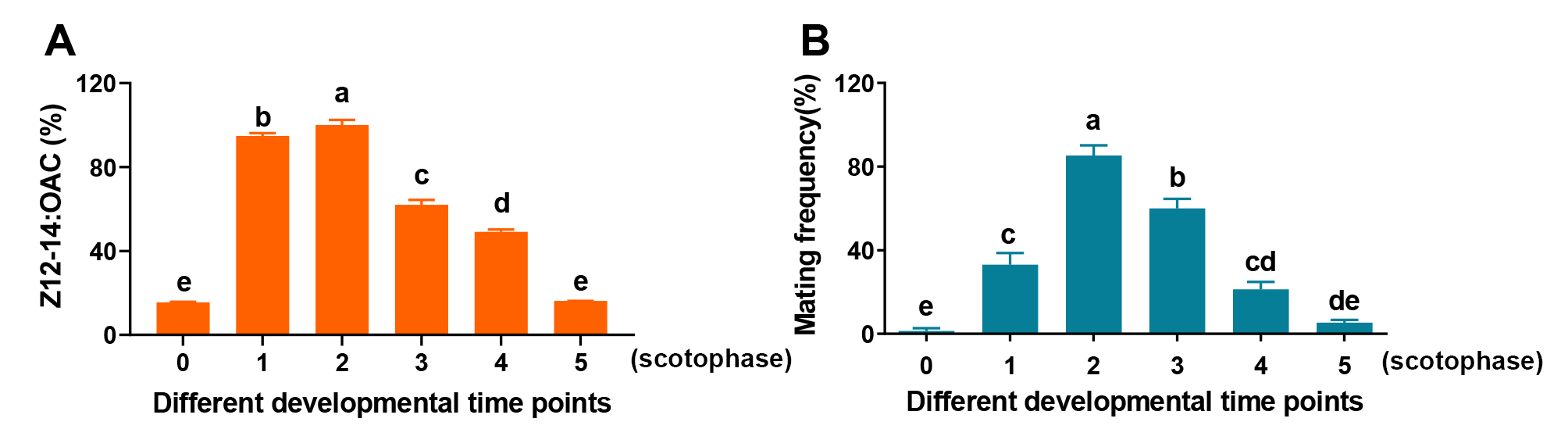

Supplement: Supplementary file 5 [file Image_4.tif]

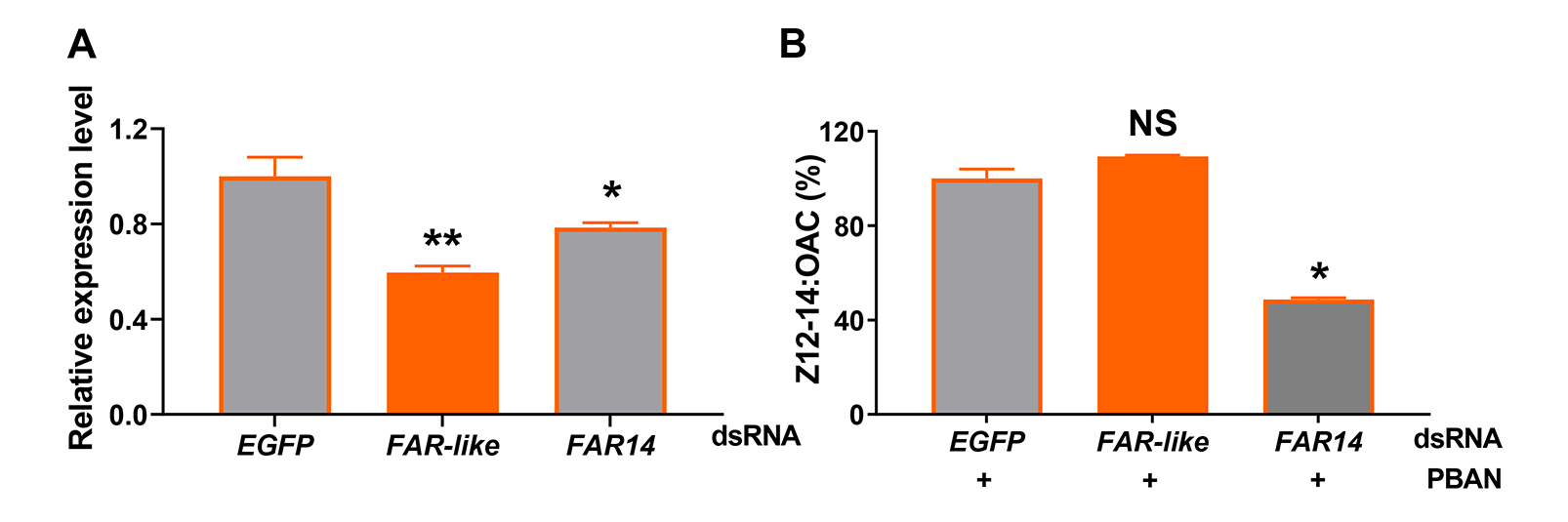

Supplement: Supplementary file 6 [file Image_5.tif]
