# Supplementary material for: Transcriptome Analysis of Ostrinia furnacalis Female Pheromone Gland: Esters Biosynthesis and Requirement for Mating Success
Source: Front Endocrinol (Lausanne). 2021 Sep 17;12:736906. doi: 10.3389/fendo.2021.736906 (PMC8485726; doi:10.3389/fendo.2021.736906)
Supplement: Supplementary file 7 [file Table_1.docx]

Table S1 Primers used in this study

| Purpose | Primer name | Primer sequence（5′→3′） | Size/bp |
| --- | --- | --- | --- |
| Real-time qPCR | PBANR-RT-F | AAATAGACAACGGGCAAACA | 180 |
|  | PBANR-RT-R | AAAGCTTCTTAATCGGGTG |  |
|  | CaN-RT-F | TGCCACCCTATTACGATT | 212 |
|  | CaN-RT-R | ACAACTTCAAAGCCCAGA |  |
|  | ACC-RT-F | GGAAACGAGGACGGTAG | 175 |
|  | ACC-RT-R | CCAGTTGGCGAAGATGA |  |
|  | PKA-RT-F | CCAACATAAACCGACGAA | 201 |
|  | PKA-RT-R | TCACCACCTGGCACATAC |  |
|  | PKCδ-RT-F | GAAGTACAACCAGGCGGTAG | 193 |
|  | PKCδ-RT-R | CGCATCTTTGTCCAACAACC |  |
|  | FAR-RT-F | TCCACTGACATGGGAGAAGT | 171 |
|  | FAR-RT-R | GCGATTCCGTCGATGATGTA |  |
|  | FAR14-RT-F | AAACAAGCTTGAAAAGGCCG | 199 |
|  | FAR14-RT-R | TTTGAACAGGAACCTGCGAA |  |
|  | △14L-RT-F | TGACAAGCCAGAGGAC | 193 |
|  | △14L-RT-R | CACCAGCGAGTAGAAGA |  |
|  | 18s-RT-F | CAACACGGGAAATCTCACCA | 190 |
|  | 18s-RT-R | AAATCGCTCCACCAACTAAG |  |
|  | β-actin-RT-F | CCTCCACCTCCTCGAGAAG | 185 |
|  | β-actin-RT-R | GGCAACGGAACCTCTCGTTA |  |
| RNAi | PBANR-T7-F | GCGTAATACGACTCACTATAGGGAATGGAGAGCAGCGAGC | 521 |
|  | PBANR-T7-R | GCGTAATACGACTCACTATAGGGCGCTCGGAGACTCGCTC |  |
|  | CaN-T7-F | GCGTAATACGACTCACTATAGGGGGAGGCATTTGATTGCCTAC | 488 |
|  | CaN-T7-R | GCGTAATACGACTCACTATAGGGAGTACGGGTGAGGTGAACAA |  |
|  | ACC-T7-F | GCGTAATACGACTCACTATAGGGCGATCTACCACGAGGTCG | 458 |
|  | ACC-T7-R | GCGTAATACGACTCACTATAGGGCTGGTCCAAGTCTAATGA |  |
|  | FAR-T7-F | GCGTAATACGACTCACTATAGGGATACAGGAACAATTTGATG | 539 |
|  | FAR-T7-R | GCGTAATACGACTCACTATAGGGGTTGCAATAACAATAGCGG |  |
|  | FAR14-T7-F | GCGTAATACGACTCACTATAGGGGGCTATGGCGAGGAGGCG | 524 |
|  | FAR14-T7-R | GCGTAATACGACTCACTATAGGGGCTGCCACGTGATGGGGT |  |
|  | EGFP-T7-F | GATCACTAATACGACTCACTATAGGGAGACCTGAAGTTCATCTGCACCAC | 485 |
|  | EGFP-T7-R | GATCACTAATACGACTCACTATAGGGAGACTCCAGCAGGACCATGTGATC |  |
