# Supplementary material for: Transcriptome Analysis of Ostrinia furnacalis Female Pheromone Gland: Esters Biosynthesis and Requirement for Mating Success
Source: Front Endocrinol (Lausanne). 2021 Sep 17;12:736906. doi: 10.3389/fendo.2021.736906 (PMC8485726; doi:10.3389/fendo.2021.736906)
Supplement: Supplementary file 8 [file Table_2.docx]

Table S2. The number of unigenes annotated in public database searched

| DataBase | Number of Unigenes |
| --- | --- |
| Uniprot  NR  EggNog  Go  KEGG  Total | 55013  55638  49307  38473  12750  56914 |
