# Supplementary material for: Transcriptome Analysis of Ostrinia furnacalis Female Pheromone Gland: Esters Biosynthesis and Requirement for Mating Success
Source: Front Endocrinol (Lausanne). 2021 Sep 17;12:736906. doi: 10.3389/fendo.2021.736906 (PMC8485726; doi:10.3389/fendo.2021.736906)
Supplement: Supplementary file 9 [file Table_3.docx]

Table S3. Distribution of unigene and transcript size in *O. furnacalis* PG transcriptome assembly.

|  | Min Length | Mean Length | Max Length | N50 | N90 | Total Nucleotides |
| --- | --- | --- | --- | --- | --- | --- |
| Transcripts | 201 | 1066 | 32296 | 2177 | 373 | 130175659 |
| Unigenes | 201 | 814 | 32296 | 1619 | 298 | 63133438 |
