# Supplementary material for: Transcriptome Analysis of Ostrinia furnacalis Female Pheromone Gland: Esters Biosynthesis and Requirement for Mating Success
Source: Front Endocrinol (Lausanne). 2021 Sep 17;12:736906. doi: 10.3389/fendo.2021.736906 (PMC8485726; doi:10.3389/fendo.2021.736906)
Supplement: Supplementary file 10 [file Table_4.docx]

Table S4. Unigenes possibly involved in sex pheromone biosynthesis in the *O. furnacalis* PGs.

| **Unigene** | **Lengh (bp)** | **Best Blast Match** | | | | | |
| --- | --- | --- | --- | --- | --- | --- | --- |
|  |  | **Putative identification** | **Species** | **Accession Num** | **Score (bits)** | **E-value (%)** | **Identity (%)** |
| **desaturase** |  |  |  |  |  |  |  |
| TRINITY_DN22688_c2_g8 | 1484 | stearoyl-CoA desaturase 5 | *Agrotis segetum* | KJ622048.1 | 503 | 2e-137 | 77.65% |
| TRINITY_DN24141_c5_g1 | 4117 | acyl-CoA delta-14 desaturase | *Ostrinia nubilalis* | EU350086.1 | 1834 | 0 | 96.98% |
| TRINITY_DN16498_c0_g1 | 3244 | fat body acyl-CoA delta-9 desaturase | *Ostrinia nubilalis* | AF243047.1 | 2625 | 0 | 98.59% |
| TRINITY_DN19174_c1_g1 | 2367 | sphingolipid delta(4)-desaturase DES1 | *Streltzoviella insularis* | MT318056.1 | 571 | 8e-158 | 77.47% |
| TRINITY_DN22688_c2_g5 | 995 | acyl-CoA desaturase 4 | *Plutella xylostella* | XM_038119303.1 | 536 | 1e-147 | 82.02% |
| TRINITY_DN15522_c0_g1 | 1123 | *acyl-CoA Delta(11) desaturase-like* | *Amyelois transitella* | XM_013338945.1 | 726 | 0 | 79.45% |
| TRINITY_DN27653_c0_g1 | 1581 | stearoyl-CoA 9-desaturase |  | XM_007730889.1 | 156 | 6e-33 | 71.58% |
| TRINITY_DN17842_c2_g6 | 732 | delta14-desaturase | *Ostrinia nubilalis* | EF125927.1 | 599 | 1e-166 | 93.41% |
| TRINITY_DN17114_c0_g1 | 1231 | acyl-CoA Z/E11 desaturase | *Ostrinia nubilalis* | EU350084.1 | 2163 | 0 | 98.38% |
| **fatty acyl reductase (FAR)** |  |  |  |  |  |  |  |
| TRINITY_DN21986_c0_g3 | 2941 | fatty acyl reductase | *Ostrinia scapulalis* | EU817393.1 | 1011 | 0 | 97.79% |
| TRINITY_DN18602_c0_g1 | 7932 | fatty acyl reductase 14 | *Helicoverpa armigera* | MF687541.1 | 1336 | 0 | 82.36% |
| **Calcineurin (CaN)** |  |  |  |  |  |  |  |
| TRINITY_DN13118_c0_g1 | 3001 | calcineurin A | *Galleria mellonella* | XM_026901910.2 | 4521 | 0 | 99.90% |
| TRINITY_DN22298_c3_g5 | 207 | calcineurin subunit B type 2 |  |  |  |  |  |
| TRINITY_DN18057_c0_g1 | 3284 | calcineurin subunit B type 2 | *Phalera bucephala* | LR990620.1 | 204 | 5e-47 | 88.82% |
| TRINITY_DN22298_c3_g4 | 3064 | calcineurin subunit B type 2 | *Helicoverpa armigera* | XM_021325366.1 | 2950 | 0 | 91.78% |
| **protein kinase A (PKA)** |  |  |  |  |  |  |  |
| TRINITY_DN21546_c1_g8 | 6020 | cAMP-dependent protein kinase type II regulatory subunit | *Manduca sexta* | XR_003938720.2 | 1860 | 0 | 89.72% |
| TRINITY_DN15885_c0_g1 | 4822 | cAMP-dependent protein kinase catalytic subunit | *Noctua fimbriata* | LR990937.1 | 1781 | 0 | 86.36% |
| TRINITY_DN12211_c0_g1 | 1376 | cAMP-dependent protein kinase type I regulatory subunit isoform X1 | *Amyelois transitella* | XM_013335065.1 | 832 | 0 | 80.05% |
| **protein kinase c (PKC)** |  |  |  |  |  |  |  |
| TRINITY_DN19735_c1_g1 | 2065 | protein kinase C and casein kinase substrate in neurons protein 2 | *Galleria mellonella* | XM_026895920.2 | 1502 | 0 | 81.59% |
| TRINITY_DN23371_c1_g1 | 2031 | protein kinase C isoform X4 | *Helicoverpa armigera* | XM_021336201.1 | 2453 | 0 | 88.59% |
| TRINITY_DN23600_c0_g1 | 5095 | Protein kinase C-like 3 | *Bicyclus anynana* | XM_024080517.1 | 1179 | 0 | 90.72% |
| TRINITY_DN23388_c0_g3 | 9376 | protein kinase C, brain isozyme | *Manduca sexta* | XM_030167965.2 | 2398 | 0 | 85.77% |
| **calmodulin** |  |  |  |  |  |  |  |
| TRINITY_DN21798_c4_g1 | 1381 | calmodulin isoform X1 | *Schrankia costaestrigalis* | FR997835.1 | 156 | 5e-33 | 95.83% |
| TRINITY_DN18874_c1_g6 | 969 | calmodulin-like protein 4 | *Helicoverpa armigera* | XM_021344293.1 | 390 | 1e-103 | 85.06% |
| TRINITY_DN20068_c1_g1 | 4370 | calmodulin-A isoform X1 | *Spodoptera frugiperda* | XM_035579811.1 | 1496 | 0 | 92.53% |
| TRINITY_DN17733_c0_g1 | 1876 | calmodulin-like isoform X1 | *Helicoverpa armigera* | XM_021333811.1 | 725 | 0 | 82.50% |
| **elongation of very long chain fatty acids protein** |  |  |  |  |  |  |  |
| TRINITY_DN18416_c0_g2 | 2358 | Elongation of very long chain fatty acids protein AAEL008004 | *Plutella xylostella* | XM_011552465.2 | 795 | 0 | 85.06% |
| TRINITY_DN22537_c0_g1 | 891 | elongation of very long chain fatty acids protein AAEL008004-like | *Frankliniella occidentalis* | XM_026426590.1 | 76.8 | 3e-09 | 7% |
| TRINITY_DN21888_c3_g1 | 1160 | elongation of very long chain fatty acids protein 4-like isoform X1 | *Deilephila porcellus* | LR999983.1 | 95.3 | 1e-14 | 7% |
| TRINITY_DN18120_c0_g1 | 3248 | elongation of very long chain fatty acids protein AAEL008004-like | *Helicoverpa armigera* | XM_021333313.1 | 822 | 0 | 82.19% |
| TRINITY_DN23837_c1_g6 | 2279 | elongation of very long chain fatty acids protein 7-like | *Bicyclus anynana* | XM_024079849.1 | 695 | 0 | 82.66% |
| 82.66TRINITY_DN18416_c0_g1 | 3208 | Elongation of very long chain fatty acids protein AAEL008004 | *Pararge aegeria* | XM_039893044.1 | 553 | 4e-152 | 87.90% |
| TRINITY_DN18124_c0_g1 | 2994 | elongation of very long chain fatty acids protein 7-like | *Trichoplusia ni* | XM_026873943.1 | 780 | 0 | 27% |
| TRINITY_DN22767_c0_g1 | 4726 | elongation of very long chain fatty acids protein 6 | *Amyelois transitella* | XM_013345680.1 | 760 | 0 | 79.54% |
| **fatty acid transport protein** |  |  |  |  |  |  |  |
| TRINITY_DN19613_c2_g2 | 2476 | long-chain fatty acid transport protein 4-like | *Amyelois transitella* | XM_013345203.1 | 976 | 0 | 76.86% |
| TRINITY_DN18418_c0_g1 | 2164 | fatty acid transport protein, partial | *Ostrinia scapulalis* | AB561866.1 | 2726 | 0 | 97.73% |
| TRINITY_DN22478_c0_g6 | 3999 | FATP | *Amyelois transitella* | XM_013338977.1 | 1027 | 0 | 76.86% |
| **acyl-coA binding protein** |  |  |  |  |  |  |  |
| TRINITY_DN17483_c0_g1 | 1913 | Acyl-CoA binding protein | *Deilephila porcellus* | LR999988.1 | 883 | 0 | 76.12% |
| **acetyl-coa carboxylase** |  |  |  |  |  |  |  |
| TRINITY_DN23900_c2_g1 | 5592 | acetyl-CoA carboxylase | *Heortia vitessoides* | MH246984.1 | 4370 | 0 | 81.91% |
| TRINITY_DN24168_c5_g1 | 2109 | acetyl-CoA carboxylase | *Spodoptera frugiperda* | XM_035600523.1 | 1147 | 0 | 78.45% |
| **pheromone biosynthesis activating neuropeptide receptor** |  |  |  |  |  |  |  |
| TRINITY_DN20740_c1_g3 | 2672 | pheromone biosynthesis activating neuropeptide receptor isoform B | *Ostrinia nubilalis* | JX500422.1 | 2074 | 0 | 99.13% |
| **Stim: stromal interaction molecule** |  |  |  |  |  |  |  |
| TRINITY_DN21804_c1_g1 | 3966 | stromal interaction molecule homolog isoform X2 | *Trichoplusia ni* | XM_026880382.1 | 1308 | 0 | 81.12% |
| **Acetyltransferase (ACT)** |  |  |  |  |  |  |  |
| TRINITY_DN24082_c0_g3 | 3033 | acetyltransferase 9 | *Manduca sexta* | XM_030179761.2 | 1204 | 0 | 42.60% |
| TRINITY_DN20845_c0_g1 | 2745 | acetyltransferase | *Spodoptera frugiperda* | XM_035594756.1 | 1297 | 0 | 78.21% |
| TRINITY_DN18290_c4_g4 | 2558 | acetyltransferase 4 | *Helicoverpa assulta* | MF687642.1 | 1989 | 0 | 69.55% |
| TRINITY_DN16522_c0_g1 | 1032 | Acetyltransferase 16 | *Ostrinia furnacalis* | XM_028314596.1 | 1848 | 0 | 98.50% |
| TRINITY_DN15364_c0_g1 | 1585 | acetyltransferase | *Ostrinia furnacalis* | XM_028323354.1 | 2807 | 0 | 97.28% |
